# Intracoronary epinephrine in the treatment of refractory no-reflow after primary percutaneous coronary intervention: a retrospective study

**DOI:** 10.1186/s12872-015-0004-6

**Published:** 2015-02-19

**Authors:** Tolga Aksu, Tumer Erdem Guler, Ayse Colak, Erkan Baysal, Mine Durukan, Taner Sen, Umit Guray

**Affiliations:** Department of Cardiology, Derince Education and Research Hospital, Derince, Turkey; Department of Cardiology, Ankara Yuksek Ihtisas Hospital, Ankara, Turkey; Department of Cardiology, Diyarbakir Education and Research Hospital, Diyarbakir, Turkey; Department of Cardiology, Mersin State Hospital, Mersin, Turkey; Department of Cardiology, Kutahya Evliya Celebi Education and Research Hospital, Kutahya, Turkey; Department of Cardiology, Ankara Numune Education and Research Hospital, Ankara, Turkey

**Keywords:** No-reflow, Epinephrine, Myocardial infarction

## Abstract

**Background:**

Despite the advances in medical and interventional treatment modalities, some patients develop epicardial coronary artery reperfusion but not myocardial reperfusion after primary percutaneous coronary intervention (PCI), known as no-reflow. The goal of this study was to evaluate the safety and efficacy of intracoronary epinephrine in reversing refractory no-reflow during primary PCI.

**Methods:**

A total of 248 consecutive STEMI patients who had undergone primary PCI were retrospectively evaluated. Among those, 12 patients which received intracoronary epinephrine to treat a refractory no-reflow phenomenon were evaluated. Refractory no-reflow was defined as persistent TIMI flow grade (TFG) ≤2 despite intracoronary administration of at least one other pharmacologic intervention. TFG, TIMI frame count (TFC), and TIMI myocardial perfusion grade (TMPG) were recorded before and after intracoronary epinephrine administration.

**Results:**

A mean of 333 ± 123 mcg of intracoronary epinephrine was administered. No-reflow was successfully reversed with complete restoration of TIMI 3 flow in 9 of 12 patients (75%). TFG improved from 1.33 ± 0.49 prior to epinephrine to 2.66 ± 0.65 after the treatment (p < 0.001). There was an improvement in coronary flow of at least one TFG in 11 (93%) patients, two TFG in 5 (42%) cases. TFC decreased from 56 ± 10 at the time of no-reflow to 19 ± 11 (p < 0.001). A reduction of TMPG from 0.83 ± 0.71 to 2.58 ± 0.66 was detected after epinephrine bolus (p < 0.001). Epinephrine administration was well tolerated without serious adverse hemodynamic or chronotropic effects. Intracoronary epinephrine resulted in significant but tolerable increase in heart rate (68 ± 13 to 95 ± 16 beats/min; p < 0.001) and systolic blood pressure (94 ± 18 to 140 ± 20; p < 0.001). Hypotension associated with no-reflow developed in 5 (42%) patients. During the procedure, intra-aortic balloon pump counterpulsation was required in two (17%) patients, transvenous pacing in 2 (17%) cases, and both intra-aortic balloon counterpulsation and transvenous pacing in one (8%) patients. One patient (8%) died despite all therapeutic measures.

**Conclusion:**

Intracoronary epinephrine may become an effective alternative in patients suffering refractory no-reflow following primary PCI.

## Background

No-reflow is defined as the lack of myocardial perfusion despite opening up the epicardial coronary vessels in the setting of percutaneous coronary intervention (PCI). For elective PCIs, the frequency of no-reflow can be reported as 0.6–5%. But, it may be observed in up to 50% of primary PCI cases [1,2]. The reduction of the beneficial effects of PCI is the main adverse consequence of no-reflow phenomenon [3,4]. Distal atherothrombotic embolization, ischemic or reperfusion injury, and susceptibility of coronary microcirculation to injury are held responsible for etiopathogenesis of the phenomenon [4]. Thus, pharmacologic and mechanical strategies to treat no reflow target these mechanisms. In the medical treatment of no-reflow, local vasodilator and local antiplatelet drugs have been tried extensively. Epinephrine has potent beta-2 receptor agonist properties that mediate vasodilatation of the arteriolar circulation, as well as the better known beta-1 agonist properties that increase inotropic and chronotropic stimulation of the myocardium [5,6]. Although epinephrine has been used clinically to treat cardiopulmonary arrest, there is a paucity of published data regarding its effectiveness in coronary no-reflow [7]. The potential usage of epinephrine in the treatment of no-reflow was evaluated in a previous study in which some promising results were showed [8].

The goal of this study was to evaluate the safety and efficacy of intracoronary epinephrine in reversing refractory no-reflow during primary PCI in patients with acute ST-segment elevated myocardial infarction (STEMI).

## Methods

### Patients

A total of 248 consecutive STEMI patients who underwent primary PCI between September 2009 and November 2010 at the Cardiology Department of Ankara Yuksek Ihtisas Hospital were enrolled retrospectively in the study. Eligible 12 patients who underwent primary PCI for acute STEMI within 12 hours following the onset of symptoms and received intracoronary epinephrine to reverse refractory no-reflow during primary PCI were included in the study. Acute STEMI was diagnosed on the basis of typical chest pain lasting over 30 minutes, and ST elevation of ≥1 mm in at least two contiguous ECG leads and/or ≥2 mm in precordial leads. Exclusion criterias were systolic blood pressure less than 90 mmHg at admission, a known allergic reaction to epinephrine, chronic hemodialysis, pregnancy, rescue intervention after failed thrombolysis, contraindications to aspirin or clopidogrel, need for emergent coronary artery bypass surgery, and inability to provide informed consent.

**Figure 1:**
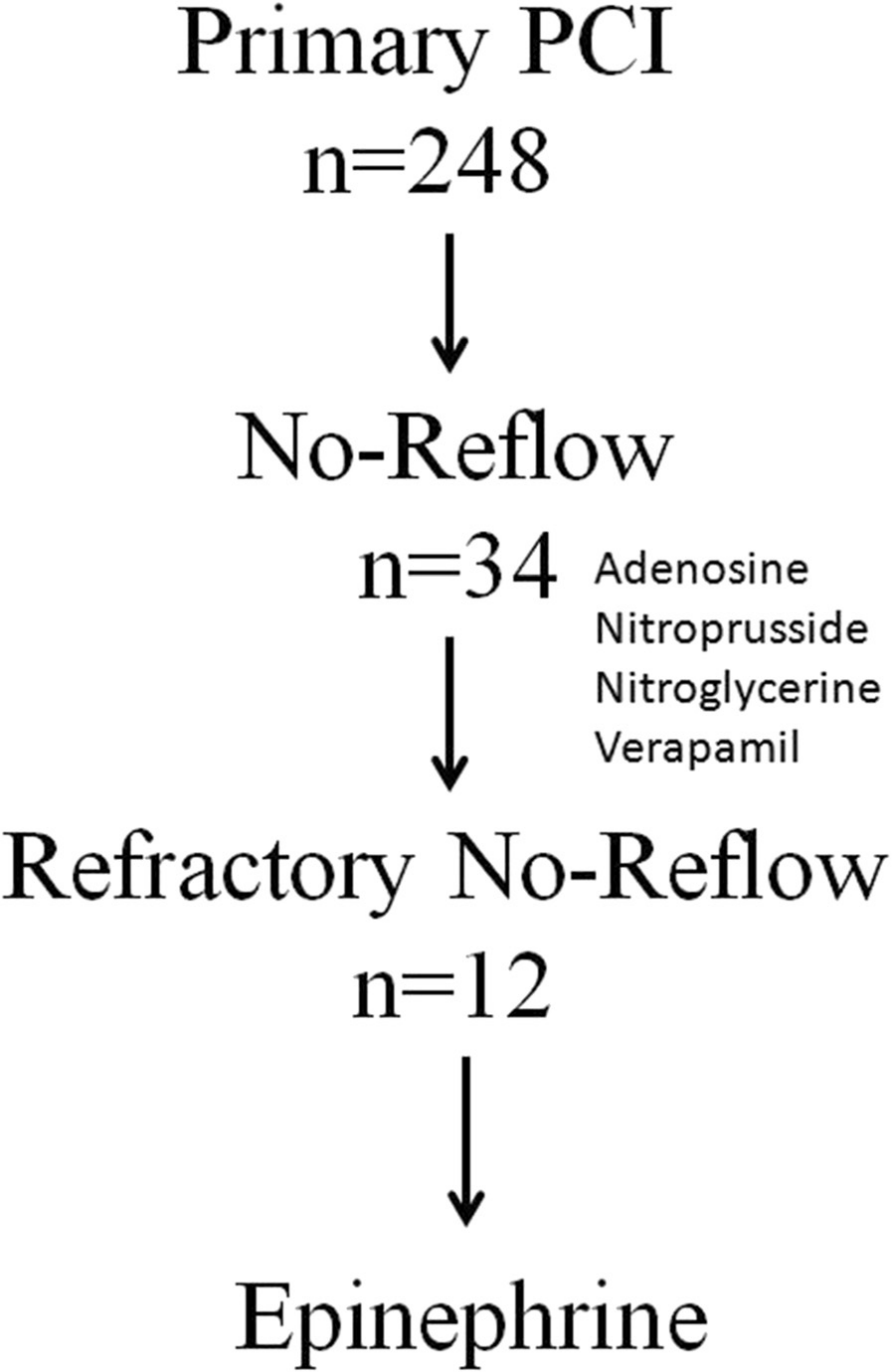
**A flowchart illustrating the selection of study patients.**

This study was approved by the Ethics Committee of the Ankara Yuksek Ihtisas Hospital, Turkey, and has been performed in accordance with the ethical standards laid down in the 1964 Declaration of Helsinki and its later amendments. All patients signed written informed consent before undergo primary PCI. All the patients provided consent upon enrolment in the study. A flowchart illustrating the selection of study patients is presented in Figure 1.

### Echocardiographic assesment

Conventional echocardiography was performed in all patients before the procedure, and at discharge (6.5 ± 4.3 days). A skilled echocardiographer blind to the clinical features of the patients performed the echocardiographic study using a Vivid 7 (GE Healthcare, Horten, Norway) ultrasound system. Basic measurements included left ventricular diameters by 2D echocardiography with settings per recommendations by the American Society of Echocardiography [9]. Left ventricular ejection fraction (LVEF) was calculated by using the biplane method (modified Simpson's rule) as recommended by the American Society of Echocardiography [10]. Echocardiograpy was not performed in 1 patient at discharge due to death of the patient.

### Angiographic assessment

All participants underwent selective coronary angiography with the Judkins technique using the Philips Angioscop Xray (Integris HM3000, Philips Medical Systems, Best, The Netherlans). Impaired blood flow was defined as a reduction in antegrade blood flow (Thrombolysis In Myocardial Infarction [TIMI]) following PCI that was not secondary to abrupt closure, spasm or significant stenosis of the original target lesion. All digital archives were taken at a speed of 25 frames/s. At least two angiographers assessed each angiogram for TIMI flow grade (TFG), TIMI myocardial blush grade (TMBG), and TIMI frame count (TFC). The two readers each graded the flows as TIMI-0, TIMI-1, TIMI-2 or TIMI-3 flow. To be considered refractory, TIMI flow ≤2 had to persist despite intracoronary administration of at least one other pharmacologic intervention (adenosine, calcium channel blocker, or nitroprusside).

All patients were subjected to oral aspirin (300 mg) and clopidogrel (300 mg), as well as intravenous 1000 U/kg unfractionated heparin. In all patients, catheterization was performed by the percutaneous femoral approach. Before the PCI, standard left and right coronary angiograms with at least 2 best projections were obtained for each patient. The effects of intracoronary epinephrine on qualitative TFG or TMBG and quantitative TFC were recorded. The time from cineangiogram documenting no-reflow to epinephrine dose and the time from epinephrine to follow-up cineangiogram were calculated.

### Hemodynamic analysis

Patients were continuously monitored during all procedures. Heart rates and blood pressures records before and after administration of epinephrine were determined. Regardless of atrial or ventricular, all tachy or bradi-arrhythmias after epinephrine were recorded and analyzed to ascertain adverse clinical effects of epinephrine on cardiac hemodynamics. Hypotension was defined as systolic blood pressure that was <90 mm Hg, or intraaortic balloon pump (IABP) in order to maintain a systolic blood pressure >90 mm Hg.

### Management of no-reflow

The first medical treatment of no-reflow was left to the discretion of the operator. All patients received intracoronary epinephrine for treatment of refractory no-reflow at a dose of at least 100 μg (range, 100–400 μg) given through the central lumen of an over-the-wire balloon catheter.

### Follow-up

The follow-up information was obtained by a telephonic interview at 4 years. Major adverse cardiac events (MACE) including re-infarction, revascularization, and death, was obtained from hospital records, death certificates, or telephone contact with relatives of the patient or referring physician.

### Endpoints

Procedural success was defined as ≤50% stenosis, TFG 3, and/or increase in TMBG at least 1 grade. Recovery of hemodynamical parameters was accepted as clinical success. The data of usage of transvenous pacemaker or IABP, hemodialysis or mechanical ventilation requirement and mortality were collected.

### Statistical analysis

Statistical analysis was performed by SPSS 11.5 statistical software. Results were presented as mean **±** standard deviation (SD) or number (%) of patients. The comparison of the data between the two groups was performed by an unpaired Student′s *t* test for continuous variables and by a chi-square test for discrete variables. Categorical variables were compared using chi-square test. *P* <0.05 was considered statistically significant.

## Results

The clinical and procedural characteristics of the patients are shown in Table 1. No-reflow was initially treated with intracoronary adenosine in 16.7% of patients (mean dose, 150 μg), nitroprusside in 16.7% (mean dose, 250 μg), nitroglycerine in 33.3% (mean dose, 200 μg), and verapamil in 33.3% (mean dose, 300 μg). After an unsuccessful attempt with one of these drugs, intracoronary epinephrine was used. The time from cineangiogram documenting no-reflow to the first epinephrine dose was 8.4 ± 3 min and the time from epinephrine to follow-up cineangiogram was 2.1 ± 1.3 min.

**Table 1:** **Baseline clinical and procedural characteristics of the patients who receiving intracoronary epinephrine**

**Characteristics**	**Rate**
Gender (% male)	8 (67%)
Age (mean ± SD)	62 ± 12
**Comorbid conditions**	
Diabetes Mellitus	2 (17%)
Hypertension	4 (33%)
Previous CABG	1 (8%)
**Lesion location**	
Left anterior descending	4 (33%)
Circumflex	2 (17%)
Right coronary artery	6 (50%)
Preprocedural angiographic appearance of thrombus*	8 (67%)
**TIMI flow grade before epinephrine**	
TIMI 1	8 (67%)
TIMI 2	4 (33%)
**TIMI flow grade after epinephrine**	
TIMI 1	1 (8%)
TIMI 2	2 (17%)
TIMI 3	9 (75%)
TIMI frame count before epinephrine (mean ± SD)	56 ± 10
TIMI frame count after epinephrine (mean ± SD)	19 ± 11

*Preprocedural thrombus was defined as discrete intraluminal filling defect, contrast staining, or haze in the target vessel or at the site of the target lesion.

**Figure 2:**
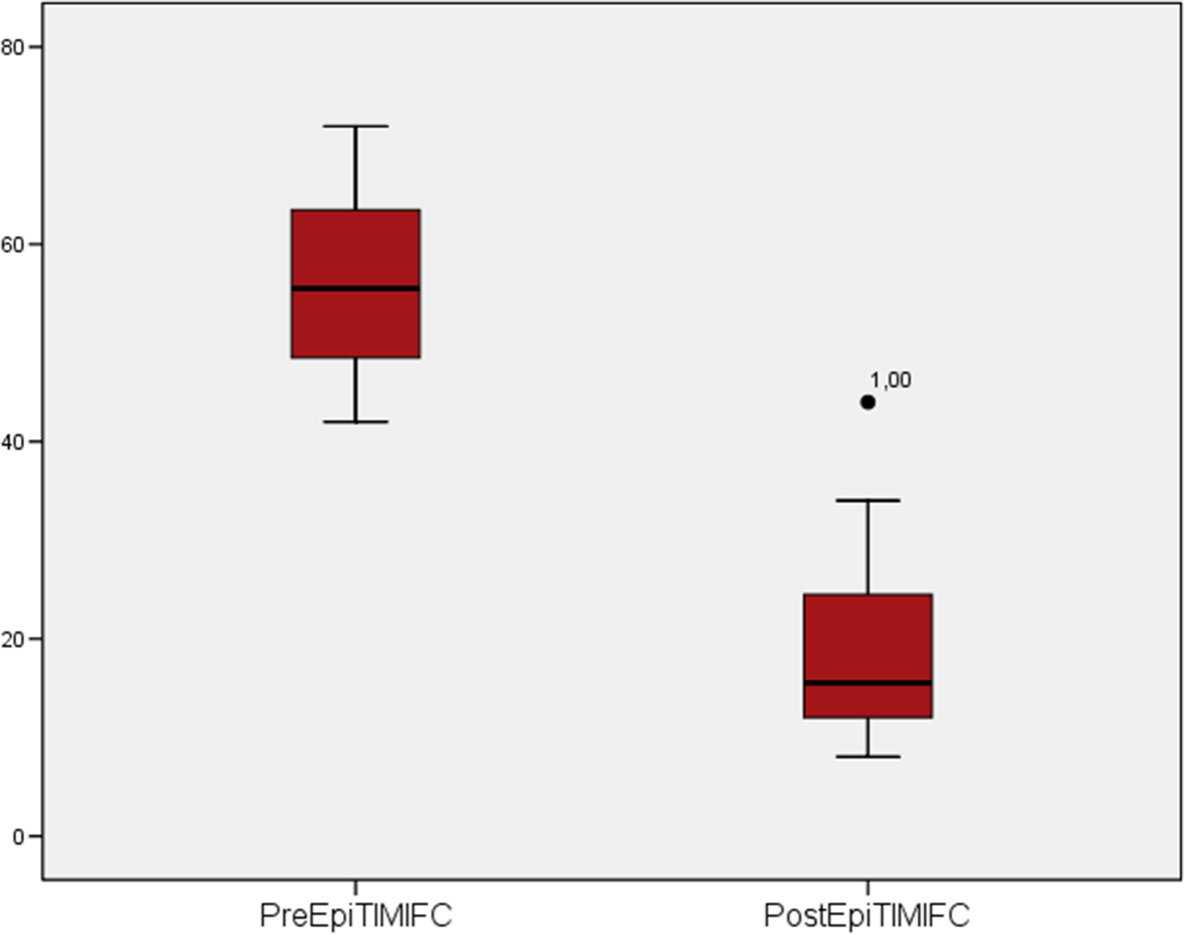
**TIMI frame count before and after intracoronary epinephrine bolus are shown.**

**Figure 3:**
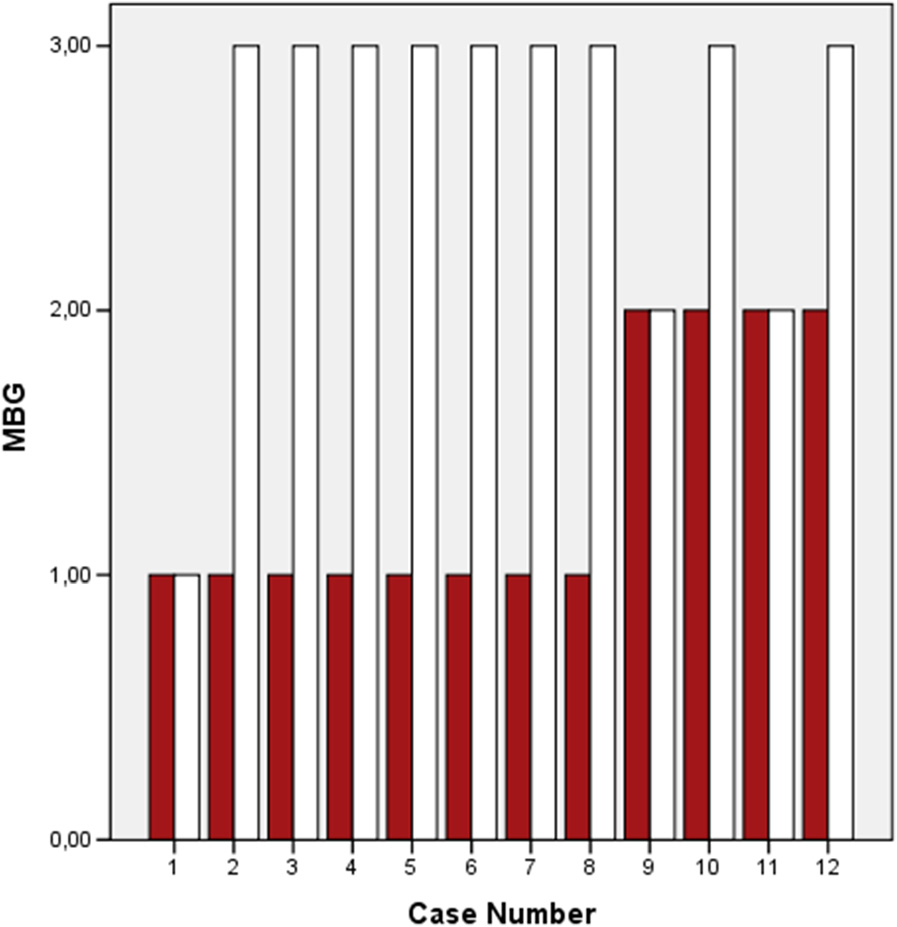
**The levels of myocardial blush grade before and after intracoronary epinephrine bolus are shown.**

Administration of intracoronary epinephrine (mean dose, 333 ± 123 μg) resulted in significant overall improvement in coronary flow grade from 1.33 ± 0.49 to 2.66 ± 0.65 (p < 0.001). No-reflow was successfully reversed with complete restoration of TFG 3 in 9 of 12 patients (75%). There was improvement in coronary flow of at least one TFG in 11 (93%) patients, two TFG in 5 (42%) cases. TFC decreased from 56 ± 10 to 19 ± 11 (p < 0.001) (Figure 2). Also, a reduction of TMBG from 0.83 ± 0.71 to 2.58 ± 0.66 was detected after epinephrine bolus (p < 0.001) (Figure 3). No-reflow phenomenon was resulted with hypotension in 5 (42%) patients. After administration of intracoronary epinephrine, hypotension was resolved all but one patient (8%). Mean systolic blood pressure was 140 ± 20 mmHg at the end of the procedure. Hemodynamic effects of intracoronary epinephrine are shown in Table 2.

**Table 2:** **Hemodynamic effects of intracoronary epinephrine**

**Predosage**	**Postdosage**
Systolic pressure (mmHg)	
94 ± 18	140 ± 20*
Diastolic pressure (mmHg)	
61 ± 14	92 ± 16*
Heart rate (beats/min)	
68 ± 13	95 ± 16*

*p < 0.001.

None of the patients complicated by sustain ventricular tachycardia. However, non-sustain ventricular tachycardia was detected in 3 (25%) patients. Atrial arrhythmia was not detected in any patients. During the procedure, IABP was required in two (17%) patients, transvenous pacing in 2 (17%) cases, and both IABP and transvenous pacing in one (8%) patients. None of the patients required hemodialysis during hospitalization period. Mechanical ventilation was required in only one patient (8%). And, this patient died 22 hours after the procedure, despite intensive medical treatment.

Baseline mean LVEF was increased significantly at discharge from 39.33 ± 6.49 to 42.09 ± 5.52 (p < 0.001). The decrease in left ventricular diameters was also significant compared baseline records. (Left ventricular end-diastolic and end-systolic diameters decreased from 50.50 ± 4.29 to 48.18 ± 3.42, and from 43.90 ± 4.57 to 41.36 ± 4.36, respectively, p <0.001).

### Follow-up

During the 4-year follow-up period, 2 patients (17%) with TFG 2 after intracoronary epinephrine underwent target lesion revascularization for STEMI; hospitalization due to heart failure occurred in 1 patient (8%). All patients were alive at the end of follow-up.

## Discussion

Our single center study demonstrated that intracoronary epinephrine may be an effective option in the patients with refractory no-reflow following primary PCI for STEMI. Intracoronary epinephrine resulted in significant improvement in TFG, TFC, and TMBG in almost all patients. Moreover, epinephrine administration was well tolerated without serious adverse hemodynamic or chronotropic effects.

The results of our study are consistent with a previous retrospective study conducted by Skelding et al. [8]. They performed a retrospective analysis of the angiographic, hemodynamic, and clinical effects of intracoronary epinephrine administered in patients developing refractory no-reflow following PCI. Of the 29 patients, 7 (24%) underwent elective PCI, and in 22 others (76%) refractory no-reflow developed following PCI in the setting of acute coronary syndromes. In their study, administration of intracoronary epinephrine resulted in significant improvement in TIMI flow in almost all patients and restored normal flow in the majority of cases. Unlike the previous study, only patients with STEMI were included in the present study.

Although the study by Skelding et al. [8] adds to our knowledge by providing first information about the safety and efficacy of intracoronary epinephrine in refractory no-reflow, there remain a number of important unresolved issues. The optimal dose, route, and frequency of administration for this agent have been shown to effectively treat no-reflow have not been known clearly yet. As we mentioned above the final cumulative dose was not predefined in the previous study. In our study, empirical starting dose of epinephrine was 100 μg and this was increased up to 400 μg based on patient’s responses.

The mean dose of intracoronary epinephrine was significantly higher in our study. Although the exact reason of this finding cannot be thoroughly understood, a possible explanation is that six (50%) of the patient necessitated multiple doses in our study. Whereas, multiple doses of intracoronary epinephrine were given in only 1 (3%) patient in the study mentioned above [8].

The definition of hypotension after no-reflow was not stated in the previous study. Although the ratio of hypotensive patients were seen similar between previous and current studies (48% and 42%, respectively), the only patients in whom systolic blood pressure decreased significantly after no-reflow phenomenon were defined as hypotension in the present study. A possible explanation of higher epinephrine administration in the current study is that the ratio of severe hypotensive cases may be higher in our study. Our study population consisted of patients with STEMI and severe hemodynamic instability may be more observed in the STEMI after no-reflow. These may explain why the patients more resistant to the single dose epinephrine administration in our study.

As another difference, the effects of intracoronary epinephrine administration patients had been evaluated only by TFG in the previous study. However, it is well known that angiographic analysis of TFG is qualitative, and it does not precisely quantities flow changes or distinguish microvascular versus myocardial mechanisms of no-reflow. As quantitative angiographic parameters, TFC can be used to describe the effectiveness of myocardial reperfusion more accurately. Furthermore, TMBG is an independent predictor of long-term mortality [11]. Our study demonstrated that administration of intracoronary epinephrine exerts improving effects in quantitative angiographic parameters (TFC ad TMBG) as well as TFG. Also, it was confirmed that selective intracoronary epinephrine administration is safe and well-tolerated.

No-reflow is a relatively common and serious complication of PCI strategies. It has been demonstrated that either impaired flow or the absence of flow is associated with an increased follow-up incidence of myocardial infarction and death [12]. The cause of no-reflow after primary PCI in patients with STEMI is multifactorial. Endothelial dysfunction, microvascular disorders, spasm, embolization, and reperfusion injury are thought to be responsible for etiopathogenesis [13]. Furthermore, advanced age, delayed reperfusion, a low TIMI flow before PCI, systolic blood pressure on admission <100 mmHg, usage of IABP before PCI, a long target lesion and a high thrombus burden were found as independent predictors of no-reflow [14]. Thus, pharmacologic strategies for the treatment of no reflow have focused primarily on two strategies: local vasodilator therapy and local antiplatelet therapy.

Among the mechanisms responsible from the no-reflow phenomenon, vasoconstriction is considered one of the most important and potentially reversible, as suggested by the numerous positive reports of therapeutic vasodilatation in this context. Epinephrine causes potent coronary vasodilator effect via β2 receptor activation, in addition to its chronotropic and inotropic effects on the heart. Thus, it should not be confusing that intracoronary epinephrine may reveal a beneficial effect on the prevention of the no-reflow in the patients with STEMI.

As a second responsible mechanism, platelet aggregation may play an important role in the formation of embolization and thus the occurring of no-reflow. Glycoprotein ΙΙb/ΙΙΙa inhibitors block the final pathway of platelet aggregation and should be effective in reducing both epicardial and microvascular thrombus burden. The previous studies revealed that early administration of Glycoprotein IIb/IIIa inhibitors before PCI may improve early infarct-related artery patency before stenting, the success rate of the stenting procedure, the rate of coronary patency at follow-up, left ventricular function, and clinical outcomes especially among those patients with preprocedural TIMI 0 or 1 flow [15-17]. However, there are no convincing randomized trials in using Glycoprotein IIbIIIa inhibitors in the treatment of no-reflow, and therefore their use for treatment of no-reflow is not a guideline recommendation.

Saito et al. [18] evaluated the effectiveness of pulse infusion thrombolysis in patients with an AMI with a large (>15 mm) coronary thrombus, focusing on the occurrence of the 'no flow' phenomenon. They found that pulse infusion thrombolysis was effective in preventing 'no flow' in the mechanical revasculalization for AMI especially those cases with a large thrombus.

Finally, microvascular obstruction (MVO) is another integral part of myocardial no-reflow. Experimental and clinical studies demonstrate that ischemic times of 3–4 hours result in irreversible microvascular injury [19]. Magnetic Resonance Imaging (MRI) is the most accurate method for the evaluation of MVO [20,21]. In a recently published study, Pernet et al. [22] showed that although 93 to 95% of the patients had a TIMI flow grade =3 after PCI at 24 h, MVO was diagnosed by MRI in 57% of cases at day 5 [19].

Although, chronotropic and inotropic effects on the heart of epinephrine are well known, epinephrine may show potent beta-2 receptor agonist properties that mediate vasodilatation of the arteriolar circulation. This may be one of the potential explanations of the beneficial effect on no-reflow phenomenon. In addition, no-reflow phenomenon is usually presented with hypotension. Intracoronary epinephrine may restore normotension in these patients, since this agent stimulates alpha vasoconstrictor receptors. The increase in coronary flow due to correction of hypotension may be the other potential mechanism. Despite the encouraging results of our study, pro-arrhythmic potential of epinephrine and the possibility of worsening ischemia by increasing myocardial oxygen demand should be explained by large randomized studies.

### Limitations

There are several limitations of our study. The number of patients is small. Also, the study was designed retrospectively and therefore subject to selection bias. Retrospective review of data also limits data regarding the quantitative responses of coronary flow and systemic hemodynamic to pharmacological interventions.

Theoretically, post-PCI angiographic parameters of coronary flow may be well if preprocedural Glycoprotein IIb/IIIa inhibitor loading was done routinely in all patients. It cannot be concluded if the patients would get loading of Glycoprotein IIb/IIIa inhibitors, whether useful effects of intracoronary epinephrine change.

At the early phase of STEMI, the successful restoration of epicardial coronary artery patency does not always predict to adequate reperfusion at the microvascular level. So, the other potential limitation of our study is that no patients were evaluated by MRI to confirm the adequate reperfusion at the microvascular level.

Lastly, the follow-up information was obtained by a telephonic interview. If all patients were evaluated by echocardiography for left ventricular remodeling at follow-up, it would help to clarify not only angiographic but also clinical effects of intracoronary epinephrine treatment.

## Conclusion

In summary, our study revealed that intracoronary epinephrine may exert encouraging effects in patients developing refractory no-reflow following primary PCI for STEMI. Intracoronary epinephrine was well tolerated and resulted in prompt and dramatic recovery of flow in the majority of patients. Prospective randomized studies will be necessary to determine whether intracoronary epinephrine should be used primarily or in combination with other agents for the treatment of no-reflow.
